# N-Acetylcysteine for the Management of Non-Acetaminophen Drug-Induced Liver Injury in Adults: A Systematic Review

**DOI:** 10.3389/fphar.2022.876868

**Published:** 2022-05-17

**Authors:** Judith Sanabria-Cabrera, Sara Tabbai, Hao Niu, Ismael Alvarez-Alvarez, Anna Licata, Einar Björnsson, Raul J. Andrade, M. Isabel Lucena

**Affiliations:** ^1^ Servicio de Farmacología Clínica, Instituto de Investigación Biomédica de Málaga-IBIMA, Hospital Universitario Virgen de la Victoria, Universidad de Málaga, Málaga, Spain; ^2^ UCICEC IBIMA, Plataforma ISCIII de soporte para la Investigación Clínica, Málaga, Spain; ^3^ Centro de Investigación Biomédica en Red de Enfermedades Hepáticas y Digestivas (CIBERehd), Madrid, Spain; ^4^ Servicio de Aparato Digestivo, Instituto de Investigación Biomédica de Màlaga-IBIMA, Hospital Universitario Virgen de la Victoria, Universidad de Màlaga, Malaga, Spain; ^5^ Medicina Interna ed Epatologia, Dipartimento di Promozione della Salute, Materno-infantile, di Medicina Interna e Specialistica di Eccellenza “G. D’Alessandro,” PROMISE, Università degli Studi di Palermo, Palermo, Italy; ^6^ Department of Internal Medicine, Landspitali University Hospital, Reykjavik, Iceland; ^7^ Faculty of Medicine, University of Iceland, Reykjavik, Iceland

**Keywords:** N-acetylcysteine, DILI, non-acetaminophen, acute liver injury, acute liver failure

## Abstract

**Introduction:** Idiosyncratic drug-induced liver injury (DILI) is a rare adverse reaction to drugs and other xenobiotics. DILI has different grades of severity and may lead to acute liver failure (ALF), for which there is no effective therapy. N-acetylcysteine (NAC) has been occasionally tested for the treatment of non-acetaminophen drug-induced ALF. However, limited evidence for its efficacy and safety is currently available. Our aim was to elucidate the benefit and safety of NAC in DILI and evaluate its hepatoprotective effect.

**Methods:** We conducted a systematic review to evaluate the management and prevention focused on NAC in idiosyncratic DILI. The main outcomes included mortality due to DILI, time to normalization of liver biochemistry, transplant-free survival, and adverse events. We included clinical trials and observational studies, either prospective or retrospective.

**Results:** A total of 11 studies were included after literature screening. All studies had different methodologies, and some of them had important risk of bias that may lead to interpreting their findings with caution. The majority of the studies proved NAC efficacy in a cohort of patients with ALF due to different etiologies, where DILI represented a subgroup. NAC seemed to improve transplant-free survival; however, its benefit was inconclusive in terms of overall survival. With regard to safety, NAC showed an adequate safety profile. In prevention studies, NAC showed a possible hepatoprotective effect; however, this finding is limited by the lack of studies and presence of bias.

**Conclusion:** NAC treatment seems to have some benefit in non-acetaminophen drug-induced liver failure patients with acceptable safety; however, due to the lack of evidence and limitations detected across studies, its benefit must be corroborated in clinical trials with adequate methodology.

## 1 Introduction

Idiosyncratic (unpredictable, specific to an individual) drug-induced liver injury (DILI) is a rare adverse reaction to drugs and other xenobiotics. It is considered a challenging liver disorder due to the absence of specific diagnostic biomarkers and its wide range of clinical presentations, which in some cases may lead to acute liver failure (ALF). The most important initial step in terms of management of suspected DILI is to discontinue the suspected drug. In the majority of cases, spontaneous recovery occurs, without the need for any treatment. However, in a small group of patients, worsening of injury can occur despite withdrawal of the culprit drug and can even lead to ALF (Robles-Diaz et al., 2014).

Despite some treatments being proposed as alternative therapies with variable levels of proven efficacy (European Association for the Study of the Liver, 2019), to date, none of these have been subjected to appropriate clinical research investigations in the setting of a clinical trial. Some of these treatments are used for specific drug damage, for example, the use of cholestyramine for terbinafine-induced hepatotoxicity (Mallat et al., 1997) or carnitine for valproate-induce hepatotoxicity (Bohan et al., 2001; Lheureux and Hantson, 2009). Other treatments have been recommended for specific circumstances, for example, ursodeoxycholic acid in chronic cholestasis following DILI. However, its use is controversial and not well documented (Katsinelos et al., 2000; Wree et al., 2011). The use of N-acetylcysteine (NAC) in patients with early stage ALF has been reported, but this recommendation was extrapolated from a single clinical trial performed with ALF patients due to different causes, including DILI (Lee et al., 2009).

The use of NAC to treat acetaminophen-induced liver injury (intrinsic damage) is well documented, and it is therefore commonly used to reduce severity in liver injury caused by acetaminophen overdose (Chiew et al., 2018). However, its benefit in idiosyncratic DILI is not well established. Most of the available data are based on published cases series or cohort studies with variable findings that do not allow drawing firm conclusions with regard to the benefit of NAC for idiosyncratic DILI. Very few clinical trials testing the benefit of NAC on DILI have been published (Lee et al., 2009; Nabi et al., 2017). In these studies, the cohorts consisted of ALF patients of various etiologies, where DILI patients only represented a small group. In order to clarify the role of NAC in the course of non-acetaminophen-induced liver injury in 2015, Hu et al. (2015) published a meta-analysis with information available from four clinical trials and observational studies in adults and children. They found significant differences in both native liver and post-transplantation survival between the NAC and the control group, though no differences were detected for overall survival. In a more recent meta-analysis published by Niu et al. (2021) including two clinical trials, no differences were found in terms of overall survival between patients who received NAC and the control group.

The aim of this review was to provide updated information and clarify the benefit and safety of NAC for treatment or prevention of non-acetaminophen DILI. In addition, we aimed to ascertain the best dose and duration of NAC treatment.

## 2 Materials and Methods

This systematic review was performed following the PRISMA 2020 guidelines (Page et al., 2021). Eligible literature published up to 31 September 2021 was identified through a search in PubMed, MEDLINE, EMBASE, and Web of Science. The search strategy comprised the following terms and Boolean operators (“liver injury” OR “hepatotoxicity” OR “liver failure” OR “hepatic”) AND (“acetylcysteine” OR “NAC”), with no language limitations.

The inclusion criteria for relevant studies exploring NAC in DILI prevention and treatment were original studies in adults (≥18 years) diagnosed with idiosyncratic DILI by drugs, herbal, or dietary supplements. The exclusion criteria were case reports, reviews and meta-analysis, acetaminophen overdose cases, animal models, and pre-clinical experimental studies with NAC in DILI and acute liver injury due to other etiologies.

The literature was managed using the Rayyan tool (Ouzzani et al., 2016). References cited by the included studies and reviews were reviewed to retrieve additional references. Each article was independently screened for eligibility by three different authors (JSC, ST, and RJA) based on the criteria described previously. Discrepancies were resolved by majority opinion. The work was supervised by a senior investigator (MIL).

The following data were extracted from the included publications: surname of the first author, study design, year of publication, number of patients, drug or herb responsible for DILI, DILI severity, NAC treatment regimen, outcomes, adverse events, and DILI and severity criteria when provided.

The review comprised clinical trials and observational studies with NAC administration in DILI, therapeutic outcomes (positive or negative), safety of NAC, and prevention. In addition, any other relevant and specific issue related to NAC administration in DILI was considered.

The findings from the included studies were summarized in a narrative synthesis and classified based on the study design and if NAC was used to prevent or manage DILI.

## 3 Results

**FIGURE 1:**
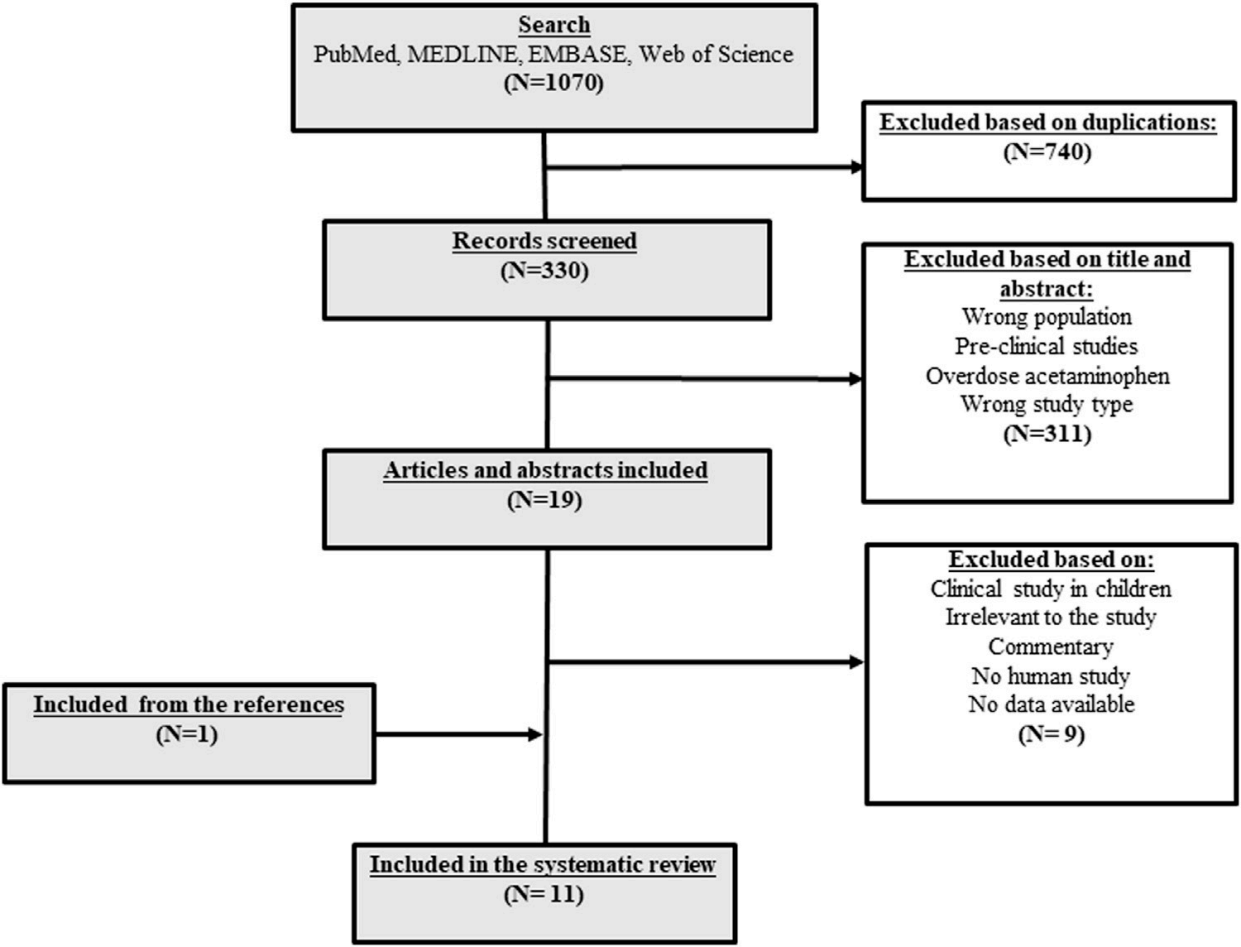
Flowchart of the search of publications about the role of NAC in DILI treatment and prevention.

A total of 1,070 studies were retrieved on the database search. Of them, 740 were duplicate records. After screening the title and abstract, 311 records did not meet the inclusion criteria and were excluded, mainly due to being irrelevant records. Thus, 19 studies were reviewed. Of them, nine records were not eligible and were excluded, mainly due to irrelevant data. After reviewing the references of the included studies, reviews, and meta-analysis identified in the literature search, an additional study was retrieved. Finally, a total of 11 studies were included (Figure 1). The studies that evaluated NAC as treatment of non-acetaminophen DILI included both randomized clinical trials (RCTs) and observational studies. The clinical trials were double-blinded (Lee et al., 2009; Moosa et al., 2021) or unknown blinded (Nabi et al., 2017), and all of them were placebo-controlled (Lee et al., 2009; Nabi et al., 2017; Moosa et al., 2021). In the majority of studies, the cohorts were formed by patients with ALF from a variety of different causes (Lee et al., 2009; Mumtaz et al., 2009; Darweesh et al., 2017; Nabi et al., 2017; Bass et al., 2021), except for two studies conducted by Moosa et al. (2021) and Borlak et al. (2018), in which the overall cohort was formed by DILI patients. In the RCTs, the total number of patients with non-acetaminophen-induced ALF (NAI-ALF) was 350, of whom 162 were DILI patients. The largest cohort of NAI-ALF patients was included in a double-blinded placebo-controlled multicenter clinical trial conducted by Lee et al. (2009). Two studies (Mumtaz et al., 2009; Nabi et al., 2017) were performed in non-liver transplantation centers.

The prevention studies included similarly both RCT and observational studies. All studies evaluated the effect of NAC in patients on antituberculosis treatment (ATT). In the RCT, the number of included patients was 273, of whom 135 were exposed to NAC. None of these studies included a placebo control arm. All prevention studies used the same NAC dose (600 mg twice daily). However, the treatment duration differed between the studies from 2 to 8 weeks.

The case definition of ALF varied across the studies and included encephalopathy with accompanying coagulopathy in the study by Lee et al. (2009) or coagulopathy with or without encephalopathy in the study by Darweesh et al. (2017). Moreover, the case definition of DILI and causality assessment was not mentioned in any of the studies except for that of Borlak et al. (2018). The route of NAC administration was intravenous and oral, alone or in combination with steroids.

### 3.1 N-Acetylcysteine Clinical Trials in Idiosyncratic Drug-Induced Liver Injury

#### 3.1.1 Treatment With N-Acetylcysteine: Efficacy and Safety

A total of five studies with data from clinical trials have been published (Lee et al., 2009; Singh et al., 2013; Stravitz et al., 2013; Nabi et al., 2017; Moosa et al., 2021). A full description of each study is presented in Table 1, including information on severity and the DILI criteria applied when provided. The studies published by Lee et al. (2009), Nabi et al. (2017), and Moosa et al. (2021) were original RCTs, while those published by Singh et al. (2013) and Stravitz et al. (2013) were sub-studies based on an earlier published study (Lee et al., 2009). It is important to highlight that the studies published by Lee et al. (2009) and Nabi et al. (2017) evaluated the effect of NAC in a cohort of ALF due to different etiologies, where DILI patients only represent a subgroup.

**TABLE 1:** Characteristics of randomized clinical trials included in the systematic review.

Study, year (country)	Clinical trial design	Age (y)	Patients (Ex/Cn)^a^	Treatment regimen	Outcomes primary (P) secondary (S)	TD	DILI criteria/severity	Events^d^ (Ex/Cn)	AE (Ex/Cn)	Summary of conclusions
DILI prevention										
Baniasadi et al. (2010) (Iran)	Study type: interventional	≥60	28/32	Ex: ATT plus NAC 600 mg (b.d)	P: ATT-induced DILI incidence	2 weeks	DILI criteria (at least one): (i) ALT and/or AST > 5xULN. (ii) TBil > 1.5 mg/dl. (iii) Any increase in ALT and/or AST along with hepatitis symptoms	0/12	NA	NAC protects against ATT-induced DILI
	Randomization: NA									
	Allocation: NA									
	Open-label			Cn: ATT alone						
	SSE: 52									
	ITT: NA						Severity: NA			
Thandassery et al. (2012) (Taiwan)	Study type: interventional simple	NA	26/25	Ex: ATT plus NAC 600 mg (b.d)	P: DILI severity (TBil and transaminases), time to normalization of liver profile, oxidative stress parameters	8 weeks	DILI criteria: NA	8/8^f^	NA	NAC showed no effect on DILI severity, biochemical recovery, and oxidative stress in cases with ATT-induced DILI
	Randomization-Allocation: NA						Severity: (i) TBil > 5 mg/dl (ii) Transaminases > 10xULN			
				Cn: ATT alone						
	Open-label									
	SSE: NA									
	ITT: NA									
Ahmed and Rao (2020) (Pakistan)	Study type: interventional	18–60	81/81	Ex: ATT plus NAC 600 mg (b.d)	P: ATT-induced DILI incidence	2 m	NA	1/14	NA	NAC is effective in preventing ATT-induced DILI
	Simple randomization: NA									
	Allocation: NA			Cn: ATT alone						
	Single-blind									
	SSE: 169									
	ITT: NA									
DILI Treatment										
Lee et al. 2009 (United States)	Study type: interventional	≥18	19/26^e^	Ex: 5% GCL with NAC (150 mg/kg/h Over 1 h, 12.5 mg/kg/h for 4 h, 6.25 mg/kg/h for 67 h)	P: Overall survival at 3 weeks	72 h	DILI criteria: NA	15/17	46/NA	NAC improves transplant-free survival in early-stage NAI-ALF
	block									
	Randomization: yes						Severity: ALF: any degree of encephalopathy and INR ≥ 1.5 due to an illness of < 24 weeks			
	Allocation: yes			Cn: 5% GCL	S: Transplant-free survival at 3 weeks, LT					
	Double-blind									
	SSE: 170									
	ITT: Yes									
Singh et al. 2013 ^c^ (United States)	Study type: interventional block	≥18	81/92	Ex: 5% GCL with NAC (150 mg/kg/h over 1 h, 12.5 mg/kg/h for 4 h, 6.25 mg/kg/h for 67 h)	P: LT and death	72 h	DILI criteria: NA	NA	NA	Decreased risk of LT/death or LT alone with NAC in early coma grade NAI-ALF patients was reflected in improved ALT and TBil, but not in INR, creatinine, or AST
	Randomization: yes									
	Allocation: yes						Severity: ALF: any degree of encephalopathy and INR ≥ 1.5 due to an illness of < 24 weeks			
	Double-blind	<70		Cn: 5% GCL						
	SSE:173									
	ITT: NA									
Stravitz et al. (2013) ^c^ (United States)	Study type: interventional Randomization: yes	≥18	39/39	Ex: 5% GCL with NAC (150 mg/kg over 1 h, 12.5 mg/kg/h for 4 h, 6.25 mg/kg/h for 67 h)	P: TBil and IL-17 levels	72 h	DILI criteria: NA	NA	NA	NAC may improve transplant-free survival by ameliorating the production of IL-17 in NAI-ALF
	Allocation: yes									
	Double-blind			Cn: 5% GCL			Severity: ALF: any degree of encephalopathy and INR ≥ 1.5 due to an illness of <24 weeks			
	SSE: NA									
	ITT: NA									
Nabi et al. 2017 (India)	Study type: interventional simple	≥18	10/5^e^	Ex: NAC (150 mg/kg over 1 h, 12.5 mg/kg/h for 4 h, 6.25 mg/kg/h for 67 h)	P: Survival rate, duration of hospital stay, AE	72 h	DILI criteria: NA	10/5^b^	0/NA	Recommend the use of NAC along with conventional treatments in patients with NAI‑ALF in non‑transplant centers
	Randomization: yes									
	Allocation: NA						Severity: ALF: INR of ≥ 1.5 and any degree of encephalopathy caused by illness of duration <8 weeks			
	Blinding: NA			Cn: 5% GCL for 72 h						
	SSE: NA									
	ITT: NA									
Moosa et al. (2021) (South Africa)	Study type: interventional block	≥18	53/49	Ex: NAC (150 mg/kg over 1 h, 50 mg/kg over 4 h and 100 mg/kg over 16 h)	P: Time for ALT to fall below 100 U/L	21 h	DILI criteria: ALT > 3xULN (hepatitis symptoms present) or ALT > 5xULN (without symptoms of hepatitis)	7.5 days/8 days	13/3	NAC did not shorten time of ALT decrease, but reduced length of hospital stay
	Randomization: yes									
	Allocation: yes									
	Double-blind			Cn: 0.9% NaCl or 5% GCL (if glucose <3.5 mmol/L)	S: Duration of hospital stay, mortality, AE		Severity: ALF: INR > 1.5 and altered mental status			
	SSE:100									
	ITT: Yes									

a Patients included in the final analysis.

b Number of drug-induced ALF patients who survived.

c These studies are substudies of Lee et al. (2009).

d Related to the principal outcome.

e Number of drug-induced ALF patients.

f Patients who presented severe DILI.

Abbreviations: AE, adverse events; ALF, acute liver failure; ALT, alanine aminotransferase; AST, aspartate aminotransferase, ATT, antituberculosis treatment; b.d., twice daily; Cn, control group; d, day; DILI, drug-induced liver injury; Ex, experimental group; h, hours; GLC, glucose; IL, interleukin; ITT, intention-to-treat analysis; LT, liver transplantation; m, months; NA, not available; NAC, N-acetylcysteine; NAI-ALF, non-acetaminophen-induced ALF; INR, international normalized ratio; SSE, sample size estimation; TBil, total bilirubin; TD, treatment duration; ULN, upper limit of normal; wk, week; y, years.

In the prospective double-blinded trial performed by Lee et al. (2009), 173 patients with ALF caused by different etiologies were included. DILI-induced ALF represented 26% of the entire cohort (*n* = 45). The patients in this study were randomly assigned to receive either intravenous NAC or dextrose (placebo) for 72 h. The overall survival after 3 weeks in the entire ALF cohort was similar to that in the NAC and placebo groups (70 vs. 66%, *p* = 0.283). On the contrary, transplant-free survival was higher in the NAC group than in the placebo group (40 vs. 27%, *p* = 0.043), especially in patients with coma grades I–II (52 vs. 30%, *p* = 0.010). In addition, liver transplantation (LT) rates were lower in the NAC group than in the placebo group (32 vs. 45%, *p* = 0.09). The DILI patients showed improved outcomes compared with those of other etiologies (hepatitis B, autoimmune hepatitis, or indeterminate etiologies). In the DILI group, transplant-free survival was 58% (95% CI 33–83%) for those receiving NAC compared to 27% (95% CI 8–46%) for those receiving placebo. As the number of participants in each group was small, the authors did not draw any firm conclusions or calculated significance in these analyses. Regarding safety, the occurrence of adverse events (AEs) in the overall cohort was similar in both the groups. However, some differences in specific adverse drug reactions (ADRs) were detected. Nausea and vomiting were more common in the NAC than in the placebo group (14 vs. 4%, *p* = 0.031). In total, there were five early discontinuations of therapy due to side effects possibly related to the drug, of which four were due to NAC. Although bronchospasm has been reported to be associated with NAC, only one patient in each treatment group experienced this symptom.

Two sub-studies have been published with data from the study by Lee et al. (2009). The sub-study published by Singh et al. (2013) demonstrated a decreased risk of LT/death or LT alone with intravenous NAC in early coma grade ALF patients and reflected an improvement in several parameters related to hepatocyte necrosis and biliary function (ALT and bilirubin) but not all parameters of liver injury (INR, creatinine, and AST). The second is the study published by Stravitz et al. (2013) in which proinflammatory cytokines that have been involved in the severity of hepatic encephalopathy or the outcome of patients with ALF (IL-1b, IL-2, IL-6, IL-10, IL-17, TNFα, or IFNγ) were evaluated in the serum of 39 patients who had received NAC and 39 patients who had received a placebo. The results showed that IL-17 was an independent predictor of poor outcome. In the NAC group, the concentration of IL-7 was lower than that of those who received the placebo during the study period (*p* = 0.042), suggesting the NAC treatment may improve the outcome by decreasing IL-17 concentration.

On the other hand, in the study by Nabi et al. (2017), a total of 80 patients diagnosed with NAI-ALF were enrolled in a center without transplantation facility. Forty patients were randomized to receive NAC infusion for 72 h, whereas the control group received placebo. Most of the patients had undetermined etiologies. Drug- or toxin-induced ALF represented the third most frequent etiology (*n* = 15, 18.75%). The participants in the NAC group presented a lower rate of mortality than those in the placebo group (28 vs. 53%, *p* = 0.023). The use of NAC was associated with shorter length of hospitalization in patients who survived (*p* = 0.002). Moreover, the rate of survival was improved by NAC (*p* = 0.025). Similar to the findings in the work of Lee et al. (2009), the subgroup of drug-induced ALF had a better outcome than that of other etiologies (survival rate was 100% in the NAC group vs. 60% in the control group, *p* = 0.049). No adverse event related to NAC treatment was reported in this study.

Recently, Moosa et al. (2021) conducted a randomized, double-blind, placebo-controlled trial to assess whether intravenous NAC hastens liver recovery in hospitalized adult patients with ATT-induced liver injury. A total of 53 participants were randomized to receive NAC and 49 to receive placebo. NAC was dosed and administered according to the regimen for acetaminophen overdose as per manufacturer-provided guidelines. No differences were found in terms of median time to ALT < 100 U/L and mortality rate between the two study arms. Differences were only found in median time to hospital discharge, which was shorter in the NAC arm (9 days; IQR 6–15) than in the placebo arm (18 days; IQR 10–25). Related to safety outcome, there were 16 AEs during treatment administration: 13 in the NAC arm and 3 in the placebo arm. The study infusion was stopped early due to AEs in five participants of the NAC group, all of whom were receiving NAC. These AEs consisted of nausea and/or vomiting (3), pain and drip site (1), and anaphylactoid reaction (1).

Clinical trials had an important limitation in the figure of the absence of appropriate DILI causality assessment. Acetaminophen overdose was not properly excluded in the two studies conducted by Lee et al. (2009) and Nabi et al. (2017). In the first study, almost 50% of patients were screened for acetaminophen overdose using laboratory analyses, while in the latter one, only clinical or historical evidence of acetaminophen overdose was recorded. Furthermore, in patients with important comorbidities or comedication with hepatotoxic potential, determining the culprit drug may be challenging as described in the study of Moosa et al. (2021), where patients were also under antiretroviral therapy. Finally, in one study (Moosa et al., 2021), viral hepatitis was not adequately ruled out in the NAC arm.

#### 3.1.2 Prevention With N-Acetylcysteine

Three RCTs evaluating the hepatoprotective effect of NAC have been published. Thandassery et al. (2012) conducted an RCT in which patients diagnosed with tuberculosis received only ATT or ATT plus NAC 600 mg twice daily for 8 weeks. Oxidative stress biomarkers (red blood cell glutathione, superoxide dismutase among others) were measured every 2 weeks from DILI diagnosis until 8 weeks. No differences in hepatoxicity severity defined as bilirubin >5 mg/dl (32 vs. 30.7%, control and experimental group, respectively), transaminases elevated >10 times (32 vs. 30.7%), or time to normalization of liver function tests (19.8 days vs. 18.07 days) were found. Similarly, no differences were detected for any of the oxidative stress biomarkers.

The open label RCT published by Baniasadi et al. (2010) included 60 patients aged 60 years or more who were treated with first-line ATT. Patients were randomized to receive daily oral ATT or daily oral ATT plus NAC (600 mg, orally, twice daily for the first 2 weeks of ATT). The outcome of interest was the development of DILI. A total of twelve patients (37.5%) with only ATT experienced DILI, whereas there were no DILI cases in the group with ATT and NAC. The mean duration of treatment before onset of hepatotoxicity was 4.67 ± 4.58 days.

Finally, Ahmed and Rao (2020) published an RCT in which 162 patients between 18 and 60 years newly diagnosed with tuberculosis were included. The patients were randomized to receive only standard ATT or standard ATT and NAC 600 mg twice daily. They were followed for 2 months. Liver injury occurred in 14 out of 81 patients (17.3%) in the control group and in 1 out of 81 patients (1.2%) in the experimental group. In contrast to Baniasadi et al. (2010), most of the liver injury in this study was noted in the second week of ATT.

In prevention studies, an important aspect to be considered when evaluating findings is the DILI diagnosis. This includes DILI criteria applied and use of causality assessment methods, which were not mentioned in any of the studies. In addition, studies presented relevant methodological issues as absence of placebo control arm, unblinded, and short follow-up period (Baniasadi et al., 2010).

### 3.2 N-Acetylcysteine Observational Studies in Idiosyncratic Drug-Induced Liver Injury

#### 3.2.1 Treatment: Efficacy and Safety

A total of our studies with data from observational studies have been published. A full description of each study is presented in Table 2.

**TABLE 2:** Characteristics of observational studies included in the systematic review.

Study, year (country)	Design	Mean age (y) (Ex/Cn)	Patients (Ex/Cn)^a^	Treatment regimen	Outcomes primary (P) secondary (S)	TD	DILI criteria/severity	Events^d^ (Ex/Cn)	AE (Ex/Cn)	Summary of conclusion
DILI prevention										
Torres-Diaz et al., 2018 ^c^ (United States)	Study type: retrospective	49	11	Ex: 600 mg oral NAC (b.d)	P: INH-induced DILI incidence	Average of 47 days	DILI criteria: NA	2	0	NAC is a safe and effective measure to prevent INH-induced DILI
	SSE: NA			Cn: uncontrolled			Severity: NA			
DILI Treatment										
Mumtaz et al. 2009 (Pakistan)	Study type: ambispective	28/38.5	47/44	Ex: Oral NAC (140 mg/kg, followed by 70 mg/kg, for a total of 17 doses, 4 h apart within 6 h of admission	P: Mortality	72 h	DILI criteria: NA	25/32	6/NA	NAC causes reduction in mortality and is safe to use in NAI-ALF patients
	SSE: 88			Cn: Patients not treated with NAC (historical controls)	S: AE and factors predicting mortality		Severity: ALF: impaired liver function tests and encephalopathy			
Darweesh et al. 2017 (Eqypt)	Study type: ambispective	34/35	85/70	Ex: Infusion of 150 mg/kg in 100 ml GLC 5% over 30 min, followed by 70 mg/kg in 500 ml GLC 5% over 4 h, then 70 mg/kg in 500 ml GLC 5% over 16 h. Continuous infusion of 150 mg/kg in 500 ml GLC 5% over 24 h (until INR < 1.3, twice), then oral 600 mg NAC/d	P: Mortality and LT	10 days (mean)	DILI criteria: NA	1/16^b^	96/NA	NAC reduces mortality, LT, encephalopathy, hospital stay, ICU admission, and other organ failures in NAI-ALF patients
	SSE: 88			Cn: Patients not treated with NAC (historical controls)	S: length of ICU stays, hospital stays, organ system failure, hepatic encephalopathy		Severity: ALF: TBil > 25 umol/L and INR > 1.5) with or without encephalopathy			
Borlak et al. 2018 (Germany)	Study type: retrospective	54/53	20/30	Ex: IV 5% GLC with NAC (10 g, 42 ml/h over 24 h × 7 days) and prednisolone (1 mg/kg/d until serum transaminases returned to normal)	P: ALT, AST and TBil levels	Average of 21 days	DILI criteria: NA.	3.03/8.41 (ALT mean value at at 2 weeks)	0	NAC/prednisolone was well tolerated and led to significant ALT, AST and INR improvements within 2 weeks
	SSE: NA			Cn: sFILI not treated with NAC (external group)			Severity: MELD score			
Torres-Diaz et al., 2018 ^c^ (United States)	Study type: retrospective	49	8	Ex: NA	P: ALT and AST levels	Variable duration	DILI criteria: NA	100 (ALT mean value at 30 days)	0	NAC is a safe and effective measure to treat INH-induced DILI
	SSE: NA			Cn: uncontrolled			Severity: NA			
Bass et al. (2021) (United States)	Study type: retrospective	52./52	13/40	Ex: IV NAC > 72 h	P: Time (d) to INR <1.3 or 1.5	Ex:5 days (median)	DILI criteria: NA	4/4 (median value)	NA	Extended duration of NAC leads to higher transplant-free survival, but does not appear to influence time to INR normalization or overall survival
	SSE: NA			Cn: IV NAC for 72 h	S: All-cause mortality and transplant-free survival at 3 weeks	Cn: 3 days	Severity: ALF: Encephalopathy and coagulopathy (INR ≥ 1.5) in the absence of chronic underlying liver disease, caused by illness of duration <24 weeks			

a Patients included in the final analysis.

b Number of patients who died.

c This study evaluated NAC as prevention and treatment in the same study.

d Related to primary outcome.

Abbreviations: AE, adverse events; ALF, acute liver failure; ALT, alanine aminotransferase; AST, aspartate aminotransferase; b.d., twice daily; Cn, control group; d, day; DILI, drug-induced liver injury; Ex, experimental group; h, hours; GLC, glucose; INH, isoniazid; IV, intravenous; INH, isoniazid; LT, liver transplantation; NA, not available; NAC, N-acetylcysteine; NAI-ALF, non-acetaminophen-induced ALF; INR, international normalized ratio; sFILI, severe flupirtine-induced liver injury; SSE, sample size estimation; TBil, total bilirubin; TD, treatment duration.

In the study by Mumtaz et al. (2009), 47 patients with NAI-ALF caused by different etiologies and given NAC orally were included prospectively. This group was compared with a historical control group (*n* = 44) that consisted of patients with NAI-ALF who attended the same hospital 3 years earlier and were not given NAC. Patients were recruited from a center without liver transplantation facility. Patients with drug-induced ALF represented 6.4% (*n* = 3) in the experimental group and 18.2% (*n* = 8) in the control group. The culprit drug in all cases was ATT. In the entire cohort, a total of 34 (37%) patients survived; 22 (47%) in the NAC group and 12 (27%) in the control group (*p* = 0.05). In a multivariable regression analysis, not receiving NAC along with age older than 40 years, prothrombin time (PT) more than 50 s, requiring mechanical ventilation, and interval between jaundice and hepatic encephalopathy were independent predictors of mortality. Specific data on patients with DILI were not provided. It is important to highlight that there were some differences among groups in baseline characteristics. The control group patients were older, had a lower level of bilirubin, and less grade of encephalopathy, predominantly Grade I and II, compared with the NAC group. Adverse effects were reported in 3 (6.3%) patients within 4 h of NAC administration. These were nonspecific maculopapular rashes in two patients, which resolved without any treatment within 24 h; 1 patient had transient bronchospasm, which responded to salbutamol nebulization.

Similarly, Darweesh et al. (2017) published an observational study, where 155 patients with NAI-ALF were recruited in a non-liver transplantation center. A total of eighty-five patients were prospectively included and given NAC and compared with an historical control group (*n* = 70), which was not given NAC. DILI represented the second most common etiology of ALF. Patients with drug-induced ALF were equally distributed between the two groups (37 vs. 40%: NAC and control group, respectively). In contrast to the study by Mumtaz et al. (2009), NAC was administered intravenously by continuous infusion, starting at the time of admission. The success rate (transplant-free survival) in the NAC group was 96.4%, whereas in the control group, 17 patients (23.3%) recovered and 53 (76.6%) did not, of whom 37 (53.3%) had a LT and 16 (23.3%) died (*p* = 0.01). The NAC group had significantly shorter hospital stay, less encephalopathy, and less gastrointestinal bleeding than the control group. Specific data on DILI patients were not provided. The high recovery and survival rate in this study compared with other studies could be explained by the inclusion of patients with less severe ALF (coagulopathy only, INR > 1.5) and the early start of intravenous NAC during ALF (day of admission) using a higher dose than in previous studies. Interestingly, AEs related to NAC infusion included prolonged cholestasis in 82 patients (96.4%) with a steady but slow decrease in bilirubin over a period of 2–3 months. This adverse reaction has not been reported in previous studies and the authors were unable to provide a plausible explanation for this finding. The second most common adverse reaction was dyspepsia in 11 (13%) patients. Fever and allergic reactions were reported in 3 (4%) patients, with the need for NAC discontinuation in two patients.

Borlak et al. (2018) evaluated the efficacy of NAC combined with oral prednisolone in 21 patients with severe flupirtine-induced liver injury (sFILI) in a retrospective study. This cohort of patients was compared with an external cohort of 30 sFILI cases (from spontaneous reports) that did not receive the combined treatment. Patients were administered NAC intravenously over 24 h for 7 days and an oral dose of 1 mg/kg prednisolone per day, with the dose tapered according to the biochemical response. The combined NAC/prednisolone treatment led to significant ALT, AST, and INR improvements within 2 weeks. However, the hyperbilirubinemia resolved slowly. A total of two patients with late start of NAC/prednisolone treatment developed hepatic encephalopathy and required LT. Based on serum biochemistries, sFILI cases resolved more rapidly (*p* < 0.01) than those of untreated sFILI patients, which included a fatal outcome case. The combined treatment was deemed as safe by the authors, but no details on drug safety were provided.

Torres-Diaz et al. (2018) conducted a retrospective observational study which aimed to evaluate the use of NAC for prevention and treatment of isoniazid-induced liver injury in the same study. A total of 19 patients, with an average age of 49 years, were included, of whom 8 received NAC as treatment. It should be pointed out that many of the patients presented with underlying liver disease (5 with cirrhosis and 2 with hepatic steatosis). In fact, 11 patients had hepatitis C and one had active hepatitis B infection. The patients had a favorable trend of liver enzymes after NAC initiation, with levels significantly improving by day 14. No side effects of NAC were documented. This study has several limitations, for example, the lack of a control group, the dose administered to patients was not specified, and the large proportion of patients with underlying liver disease that challenges the causality assessment, among others.

Finally, Bass et al. (2021) evaluated two dosing regimens of NAC intravenously in the management of NAI-ALF in an observational study. The standard duration of NAC was considered 72 h, whereas extended duration was defined as continuation beyond 72 h. In total, 53 patients were included retrospectively: 40 in the standard duration group and 13 in the extended duration group. The DILI patients represented 15% (*n* = 6) of the former group and 23% (*n* = 3) of the latter. There was no significant difference in the time taken to obtain a normal INR value (<1.5) between the two groups, though a higher proportion of patients in the extended duration group achieved INR normalization compared with the standard duration group (52.5 vs. 84.6%, *p* = 0.04). The transplant-free survival was higher with extended duration (76.9% extended vs. 41.4% standard; *p* = 0.03), and overall mortality at 3 weeks was notoriously lower in the extended duration, although not statistically significant from that of the other group (0% extended vs. 24.1% standard; *p* = 0.08). No differences in either hospital or ICU length of stay were observed between the groups. This study has important limitations. One of them is related to its retrospective character with inherent flaws of design. For example, the decision to extend the duration of NAC was based on clinical criteria and may have reflected clinical decision-making in the support of aggressive supportive care (i.e., in an effort to avoid LT based on patient factors) or even a selection bias due to observed positive response to therapy. All of these do not permit a solid conclusion to be drawn regarding the benefit of using an extended NAC regimen in NAI-ALF. Another important limitation was that the study did not provide any information on the occurrence of adverse reactions due to prolonged NAC use, which does not enable an adequate risk benefit evaluation. Hence, the risk of increased ADR with prolonged NAC use is currently unknown and needs further investigation.

Similar limitations, which can be a source of bias, were found in the observational studies. The most frequent limitation in the retrospective studies is the lack of information, such as incomplete patient medical histories seen in the study by Borlak et al. (2018). This makes it very difficult to determine if experimental and control groups are comparable with regards to baseline characteristics (liver parameters among others). In addition, in three of the studies (Mumtaz et al., 2009; Darweesh et al., 2017; Borlak et al., 2018), the control groups were formed of external patients who were not recruited at the same time as the patients in the experimental group. This increases the probability of the control patients having a different background. Finally, only two studies mentioned exclusion of acetaminophen overdose patients, although the exclusion was not based on analytical findings but merely on medical histories (Mumtaz et al., 2009; Darweesh et al., 2017).

#### 3.2.2 Prevention

In the study conducted by Torres-Diaz et al. (2018), eight patients were given NAC orally. Patients received NAC for an average of 47 days and had stable liver profile tests during treatment, except for two (18%) patients, whose liver enzymes increased more than 3 times the upper limit of normal. These two patients had underlying hepatitis C and liver cirrhosis. Nevertheless, only one required discontinuation of isoniazid. This study presented several limitations; the absence of control groups and the presence of a large proportion of patients with underlying liver disease challenges the causality assessment, among others.

## 4 Discussion

### 4.1 N-Acetylcysteine Treatment

According to our findings, NAC treatment shows an inconclusive effect in terms of overall survival in patients with non-acetaminophen drug-induced ALF. Similarly, published systematic reviews and meta-analysis in NAI-ALF patients showed the same contradictory results (Hu et al., 2015; Walayat et al., 2021). However, NAC seems to improve transplant-free survival. This finding is supported by previous studies conducted in NAI-ALF populations (Hu et al., 2015; Shrestha et al., 2021; Walayat et al., 2021). It is worth noting that overall survival is a general outcome that may be affected by external factors, mainly in patients under critical conditions; therefore, other more specific parameters, such as liver-related mortality, may be more accurate. In fact, NAC has shown initial favorable results when focusing on this outcome in included studies in the present review (Darweesh et al., 2017; Nabi et al., 2017). Despite the fact that NAC seems to have some benefit in non-acetaminophen drug-induced ALF and its effect may be due to its mechanism of action (anti-inflammatory, antioxidant, and vasodilator effects) (Harrison et al., 1991; Ntamo et al., 2021), findings are supported by few number of studies. In addition, the majority of studies included in this review presented important methodological drawbacks which preclude to reaching firm conclusions about its efficacy.

The limited and heterogeneous data from included studies hinder to assess properly the influence of relevant factors in NAC efficacy. For example, the time to NAC therapy initiation in NAI-ALF patients was recorded in very few studies (Mumtaz et al., 2009; Darweesh et al., 2017). Another important factor to consider is the NAC regimen administered. In most of the studies, similar loading doses of 140–150 mg/kg were used; however, subsequent doses varied. In spite of this limitation, it may be argued that at least an accumulative dose of 170 mg/kg administered over 72 h intravenously seems to be effective in the management of non-acetaminophen drug-induced ALF (based on only two RCTs). The use of higher doses did not show clear benefit; on the contrary, it may result in more ADRs (Mumtaz et al., 2009; Darweesh et al., 2017). In addition, an extended administration (>72 h) has not been properly evaluated. Finally, with the available information extracted from included studies, it is not possible to conclude which is the best route of administration as NAC was administered orally in only one study. All unresolved issues mentioned previously are also presented in acetaminophen overdoses, where NAC has been extensively evaluated. The optimal NAC regimen, which shows efficacy and less ADRs, is still under discussion (Chiew et al., 2018).

Overall, ADRs reported in the included studies are those commonly associated with NAC administration. However, in one study, authors reported a high prevalence of prolonged cholestasis (Darweesh et al., 2017). In the literature, one study reported hepatobiliary ADRs but specific data were not provided (Bateman et al., 2014). Further investigation with appropriate causality assessment is necessary to determinate if these are unexpected ADRs. In addition, the route of NAC administration seems to influence the ADR presentation; early discontinuation was reported only in studies where NAC was administered intravenously. Despite NAC treatment showing adequate security profile, it should not be forgotten that its administration may lead to relevant ADRs mainly when it is administered intravenously, and a proper follow-up should be conducted when a high dose is used and/or it is used for an extended period.

### 4.2 N-Acetylcysteine Prevention

The role of NAC in DILI prevention is difficult to assess due to the lack of studies and different outcomes evaluated. NAC seems to reduce the incidence of DILI, but it seems to have no effect in DILI severity in patients exposed to ATT. A further consideration is that the dose used in all the studies (1,200 mg per day) has not been justified. Thus, despite these initial data suggesting its hepatoprotective effect, the presence of relevant limitations, as absence of DILI causality assessment among studies, precludes to draw firm conclusions about its use as a prophylaxis alternative.

## 5 Strengths and Limitations

The strength of our review includes the performance of a comprehensive search. The studies eligible for this review were not limited to clinical trials, but observational studies were also included in the analysis to provide a broader vision of NAC information currently available. In addition, we have focused on both the hepatoprotective effect of NAC and its safety. To our knowledge, this is the most complete review carried out on NAC in idiosyncratic DILI.

The limitation of our findings is related to the quality of the studies included in the review and the limited data available. Information with regard to DILI causality assessment was rarely provided or incomplete.

## 6 Conclusion

NAC treatment seems to have some benefits in non-acetaminophen drug-induced liver failure patients; however, due to the lack of evidence and limitations detected across studies, its benefits must be corroborated. NAC administration showed an adequate safety profile; however, close follow-up is necessary, particularly when administered intravenously.

This review demonstrates the need for further research to clarify the role of NAC in treatment and prevention of non-acetaminophen DILI. This includes definition of best doses and treatment duration. There is a strong need for clinical trials with adequate design and sample size, including DILI cases that have undergone a rigorous causality assessment and have different grades of severity. This, however, represents a major challenge where institutional collaborations and the setting up a network of physicians will play a crucial role.

## Data Availability

The original contributions presented in the study are included in the article/Supplementary Material, further inquiries can be directed to the corresponding author.

## Abbreviations

AEs, adverse events; ADR, adverse drug reaction; ALF, acute liver failure; ALT, alanine aminotransferase; AST, aspartate aminotransferase; ATT, antituberculosis treatment; DILI, drug-induced liver injury; INR, international normalized ratio; LFT, liver function test; LT, liver transplantation; NAC, N-acetylcysteine; NAI-ALF, non-acetaminophen-induced ALF; PT, prothrombin time; RCT, randomized clinical trial.
